# An Infected Urachal Remnant in an Adult Managed Without Initial Surgical Intervention: A Case Report

**DOI:** 10.1155/criu/8809350

**Published:** 2026-11-14

**Authors:** J. Kyeremeh, A. Abdelrahman, I. Chipurovski

**Affiliations:** ^1^ Cambridge University Hospitals NHS Foundation Trust Cambridge UK nhs.uk

**Keywords:** adenocarcinoma, adult urachal remnant, midline mass, urachal anomaly

## Abstract

Urachal remnants are rare in adults and can present with varied clinical manifestations. Although often asymptomatic, they can become infected or undergo malignant transformation, necessitating timely diagnosis and management. This case highlights a 46‐year‐old man with a symptomatic infected urachal remnant presenting as a painful infraumbilical mass. Computed tomography demonstrated a lobulated midline fluid collection consistent with an infected urachal remnant. The collection subsequently ruptured spontaneously and drained through the umbilicus. The patient improved following drainage and antimicrobial treatment without requiring emergency surgical intervention. This case demonstrates that in carefully selected and clinically stable adults, emergency surgical excision may sometimes be avoided when effective drainage has occurred and there is a satisfactory response to antimicrobial therapy. However, resolution of the acute infection does not necessarily represent definitive treatment of the underlying urachal remnant. Specialist reassessment and consideration of elective excision or structured follow‐up remain important because recurrence and occult malignancy cannot be completely excluded without histological evaluation.

## 1. Introduction

The urachus is an embryonic remnant of the allantois, forming a connection between the foetal bladder and umbilicus, which typically obliterates by birth. Failure of involution can lead to persistent urachal anomalies, commonly diagnosed in infancy [1]. Adult presentations are rare and seen in approximately 0.063% of adults. These are often incidental or secondary to infection, inflammation or malignancy [2]. Although urachal remnants are well described in the paediatric population, adult presentations remain uncommon and are often diagnosed late due to non‐specific symptoms. Most published reports describe surgical excision as the standard treatment, particularly because of the potential risk of recurrent infection or malignant transformation. However, there is limited literature describing successful conservative management in adults, especially in cases where spontaneous rupture leads to clinical resolution. This creates uncertainty regarding the necessity of immediate surgical intervention in selected patients [3, 4]. The types of urachal remnants in adults are patent urachus, urachal sinus, urachal cyst and vesicourachal diverticulum [5]. Potential complications include infection, abscess, bladder perforation and urachal adenocarcinoma. Surgical excision has traditionally been advocated for symptomatic urachal remnants in adults because of concerns regarding recurrent infection and malignant transformation. Spontaneous involution or resolution has been described predominantly in paediatric populations; however, these findings should not be directly extrapolated to adults. Evidence supporting conservative management of infected urachal remnants in adults remains limited, particularly where definitive excision of the underlying remnant has not subsequently been performed [6].

This report is aimed at describing an infected urachal remnant in an adult that clinically resolved following spontaneous rupture, drainage and antimicrobial treatment without emergency surgical excision. The report also discusses the distinction between acute control of infection and definitive management of the underlying urachal remnant, including the need for subsequent reassessment, elective excision or structured surveillance. Though such cases are rare, they have the potential to progress to sepsis [7].

Recent case reports in adult populations have highlighted the variability in presentation and management strategies, emphasising the need for individualised decision‐making rather than uniform surgical excision in all cases.

## 2. Case Presentation

This case report has been prepared in accordance with the CARE 2023 guidelines for reporting surgical case reports. A 46‐year‐old male with a history of well‐controlled Type 1 diabetes mellitus presented to the emergency department with worsening lower abdominal pain and a progressively enlarging lump beneath the umbilicus for 6 days prior to presentation. He denied urinary symptoms, fever, weight loss or gastrointestinal complaints. His regular medication included isophane insulin only. He had no history of previous abdominal surgery and no known history of urachal abnormalities.

On clinical examination, the patient was haemodynamically stable and afebrile. Abdominal examination revealed a localised, tender, approximately 5 × 5 cm palpable mass beneath the umbilicus without overlying skin erythema or discharge at the time of admission. There was no guarding or peritonism. Examination of the external genitalia and hernial orifices was unremarkable.

### 2.1. Diagnostic Workup

#### 2.1.1. Laboratory Investigations

Laboratory investigations demonstrated an elevated white blood cell (WBC) count of 16.3 × 10^9^/L and a C‐reactive protein level of 322 mg/L, consistent with an inflammatory or infectious process. Renal function, liver function tests and electrolytes were within normal limits. Glycated haemoglobin (HbA1c) was 40 mmol/mol, confirming good diabetic control.

#### 2.1.2. Imaging Studies

Contrast‐enhanced computed tomography (CT) of the abdomen and pelvis was performed using standard portal venous phase imaging. This demonstrated a 5.4 × 4.8 × 6.2 cm lobulated fluid collection located in the midline, deep to the umbilicus and anterior to the bladder, with peripheral enhancement and surrounding inflammatory stranding, consistent with an infected urachal remnant or abscess (Figure 1A,B).

**Figure 1:**
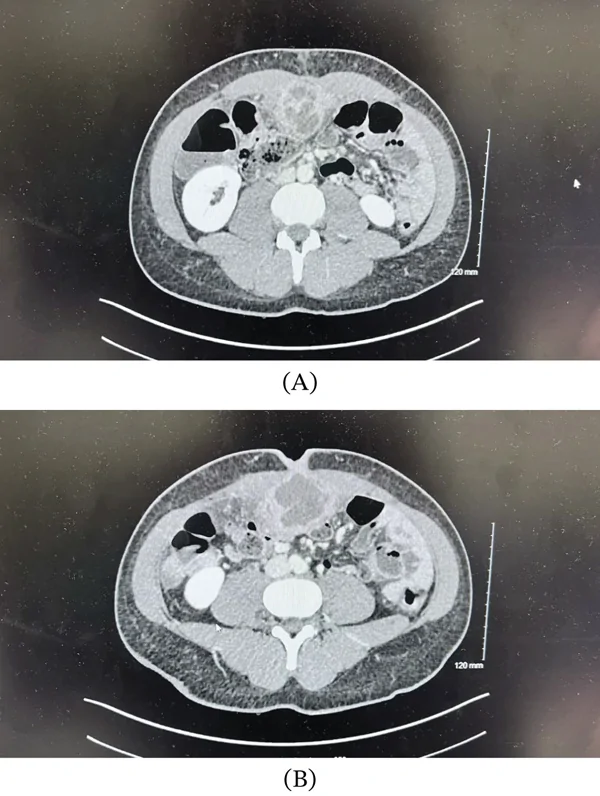
Axial contrast‐enhanced CT images demonstrating a midline cystic collection deep to the umbilicus, consistent with an infected urachal remnant. (A) Superior axial section demonstrating the midline collection. (B) Inferior axial section demonstrating the relationship of the collection to the anterior abdominal wall.

### 2.2. Management and Treatment

A conservative approach was initially adopted because of the absence of peritonitis, haemodynamic instability or radiological evidence of bladder involvement. The patient was started on empiric intravenous broad‐spectrum antibiotics while further management options, including drainage or surgical excision, were considered. He received intravenous co‐amoxiclav 1.2 grams every 8 hours according to local institutional guidelines for intra‐abdominal infection. Following spontaneous drainage of the collection and sustained clinical improvement, intravenous therapy was transitioned to oral co‐amoxiclav 625 mg every 8 hours after 3 days to complete a total antimicrobial course of 7 days. Image‐guided drainage was considered; however, this was deferred after spontaneous rupture of the collection with drainage through the umbilicus, resulting in immediate clinical improvement.

### 2.3. Microbiology Report

On the second day of admission, purulent discharge was noted from the umbilicus. Gentle expression of the area allowed drainage of residual pus. A sample of the spontaneously draining fluid was obtained through the nonsterile umbilical opening and sent for microbiological culture. This demonstrated heavy growth *of Streptococcus intermedius*, reported as sensitive to penicillin and clindamycin. Blood and urine cultures were negative, with no pathogenic organisms identified.

### 2.4. Resolution and Follow‐Up

The patient improved clinically following spontaneous drainage and antibiotic therapy. He remained afebrile after Day 2 of admission. His WBC count decreased from 16.3 × 10^9^ to 6 × 10^9^/L and a C‐reactive protein level from 322 to 38 mg/L. Total hospital stay was 3 days. He completed the prescribed course of antibiotics and remained asymptomatic at follow‐up 3 months later. The clinical course, management and follow‐up are summarised in Table 1.

**Table 1:** Timeline of clinical presentation, management and follow‐up.

Time point	Clinical event	Management/outcome
Day 0	Six‐day history of worsening lower abdominal pain and enlarging infraumbilical mass; WBC 16.3 × 10^9^/L; CRP 322 mg/L	CT demonstrated an infected urachal remnant; intravenous co‐amoxiclav commenced
Day 2	Spontaneous rupture with purulent umbilical drainage	Fluid sample obtained for microbiological culture; clinical improvement followed drainage
Day 3	Continued clinical improvement; afebrile. His WBC and CRP decreased to 6 × 10^9^/L and 38 mg/L, respectively.	Transitioned from intravenous to oral co‐amoxiclav; discharged
Day 7	Completion of antimicrobial therapy	Seven‐day total course completed
3 months	Asymptomatic at follow‐up	Safety‐net advice provided; no repeat imaging or histological assessment performed

He was advised to seek urgent medical assessment if he developed recurrent pain, umbilical discharge, fever, urinary symptoms, haematuria or a new abdominal mass. The absence of repeat imaging, histological confirmation and longer term follow‐up limits certainty regarding complete resolution of the remnant and exclusion of occult malignancy.

## 3. Discussion

Urachal remnants in adults are uncommon and frequently misdiagnosed because of their non‐specific clinical presentation. They may mimic umbilical hernia, abdominal wall abscess or bladder‐related pathology, which can delay diagnosis. Although urachal anomalies are more commonly recognised in childhood, adult cases continue to be reported, often presenting with infection, abscess formation or rarely malignant transformation [7]. These abnormalities are twice as likely in men than in women [8].

This case is notable because the acute infection resolved following spontaneous rupture, drainage and antimicrobial treatment without emergency surgical excision. However, the case does not demonstrate that antimicrobial therapy alone would have been sufficient in the absence of drainage. Spontaneous decompression was likely an important component of source control.

Most published literature recommends complete surgical removal of symptomatic urachal remnants because of the risk of recurrence or malignancy. However, some reports suggest that selected patients with spontaneous drainage and good clinical response may be managed non‐operatively with close follow‐up. This highlights the importance of individualised decision‐making rather than uniform surgical intervention in all adult cases [9].

### 3.1. Role of Type 1 DM

The patient had well‐controlled Type 1 diabetes mellitus, with a HbA1c concentration of 40 mmol/mol. Diabetes mellitus is associated with an increased risk of infection and impaired wound healing. However, the patient experienced an uncomplicated recovery following drainage and antimicrobial treatment. As this report describes a single patient, no firm conclusion can be drawn regarding the effect of diabetes on the development, severity or outcome of an infected urachal remnant. Management should therefore be based on the patient′s overall clinical condition rather than diabetes status alone [10].

### 3.2. Key Diagnostic Findings

The markedly elevated C‐reactive protein concentration (322 mg/L) and WBC count (16.3 × 10^9^/L) supported the presence of an acute inflammatory or infectious process. Imaging plays a key role in distinguishing urachal anomalies from other causes of midline abdominal masses. In children, ultrasound is often sufficient; however, in adults, CT imaging is frequently preferred because it allows better assessment of surrounding structures and helps exclude malignancy [11]. In this case, CT imaging demonstrated a midline cystic lesion anterior to the bladder, consistent with an infected urachal remnant (Figure 1A,B).

One of the main concerns in adult urachal remnants is the possibility of malignant transformation, most commonly urachal adenocarcinoma. Although this is rare, it is the primary reason why surgical excision is often recommended even after resolution of infection. In the present case, the decision to avoid immediate surgery was based on clinical improvement after spontaneous drainage and absence of suspicious features on imaging. However, the possibility of missed malignancy cannot be completely excluded without histological confirmation.

Infected urachal remnants may involve a range of aerobic and anaerobic organisms and may occasionally be polymicrobial. Reported organisms include *Staphylococcus aureus*, although other organisms such as *Escherichia coli*, *Enterococcus faecium* and *Klebsiella pneumoniae* have been documented [12]. In this patient, microbiological analysis identified *Streptococcus intermedius* from the spontaneously draining umbilical fluid which informed subsequent antimicrobial management. Because the sample was obtained through a nonsterile external opening, the culture result should be interpreted cautiously. *Streptococcus intermedius* was the predominant organism isolated, but contamination, colonisation or the presence of additional undetected organisms could not be completely excluded. The clinical response to drainage and antimicrobial treatment was therefore considered alongside the microbiology result.

### 3.3. Management Considerations

Complete surgical excision remains the generally accepted definitive treatment for symptomatic urachal remnants in adults because of the risks of recurrent infection and malignant transformation. However, surgery during active infection may be technically more difficult because of inflammation and tissue oedema. Initial control of infection using antimicrobial treatment and drainage may therefore be appropriate before elective reassessment.

In this case, spontaneous rupture resulted in drainage of the collection and was followed by rapid clinical improvement. This suggests that effective source control was central to the favourable outcome. The case does not establish that conservative treatment would have been successful if the collection had remained undrained. Patients with persistent sepsis, haemodynamic instability, peritonitis, inadequate drainage, residual abscess, worsening symptoms or failure to improve should undergo image‐guided or operative drainage [13].

The decision to avoid immediate surgery was based on the patient′s clinical stability, spontaneous drainage, clinical improvement and absence of suspicious radiological features. The benefit was therefore avoidance of emergency surgery during active inflammation, rather than proven elimination of the need for future elective treatment. This case therefore demonstrates successful initial non‐operative management of the acute infectious episode rather than definitive non‐surgical treatment of the underlying urachal remnant.

This report has several limitations. It describes a single patient, no repeat imaging was performed after treatment, histological confirmation was unavailable and follow‐up was limited to 3 months. Complete drainage of every component of the lobulated collection could not be confirmed radiologically. Occult malignancy and future recurrence could therefore not be completely excluded.

The patient was advised to seek urgent medical attention if he developed fever, worsening abdominal pain, recurrent umbilical discharge, urinary symptoms, haematuria, malaise or a recurrent abdominal mass [14].

### 3.4. Clinical Implications

Clinicians should consider an infected urachal remnant in adults presenting with unexplained lower midline or suprapubic pain, periumbilical pain, a tender infraumbilical mass, umbilical erythema or purulent discharge, recurrent umbilical infection, fever, urinary symptoms or haematuria. In this patient, the principal presenting features were lower abdominal pain and a progressively enlarging tender mass beneath the umbilicus.

Prompt imaging is important for confirming the diagnosis, assessing the anatomical extent of the lesion, identifying persistent or multiloculated collections and evaluating features that may raise concern for malignancy.

Not every clinically stable patient with an infected urachal remnant necessarily requires emergency excision when effective drainage has occurred, provided that there is a satisfactory clinical response to antimicrobial treatment. However, conservative treatment of the acute infection should not automatically be regarded as definitive treatment of the underlying remnant [15, 16].

Where excision is not performed, specialist reassessment and structured follow‐up are necessary. Follow‐up should include monitoring for recurrent abdominal or umbilical pain, umbilical discharge, recurrent infection, haematuria, urinary symptoms or a new abdominal mass. Repeat imaging should be considered if symptoms recur or if there is concern regarding persistence of the remnant or possible malignancy.

Because no histological specimen was obtained in this case, occult malignancy could not be completely excluded. The patient′s 3‐month symptom‐free follow‐up is reassuring but is insufficient to determine the long‐term risk of recurrence or malignant transformation [17].

## 4. Conclusion

Urachal remnants, although uncommon in adults, should be considered in patients presenting with lower midline abdominal pain, an infraumbilical mass, umbilical discharge or evidence of infection. CT is valuable for confirming the diagnosis, defining the extent of the collection and assessing for bladder involvement or suspicious malignant features.

This case demonstrates that emergency surgical excision may sometimes be avoided in a clinically stable adult when an infected urachal collection drains effectively and is followed by a satisfactory response to antimicrobial treatment. Spontaneous drainage was likely central to source control, and this case does not establish that antimicrobial therapy alone would be sufficient for an undrained abscess.

Clinical resolution of the acute infection should not automatically be interpreted as definitive resolution of the underlying urachal remnant. Specialist reassessment, consideration of elective excision and structured long‐term follow‐up remain important because recurrence and occult malignancy cannot be completely excluded without repeat imaging and histological evaluation.

## Funding

No funding was received for this manuscript.

## Consent

Written informed consent was obtained from the patient for the publication of this case report and any accompanying clinical images.

## Conflicts of Interest

The authors declare no conflicts of interest.

## Data Availability

Data sharing is not applicable to this article as no datasets were generated or analysed during the current study.
